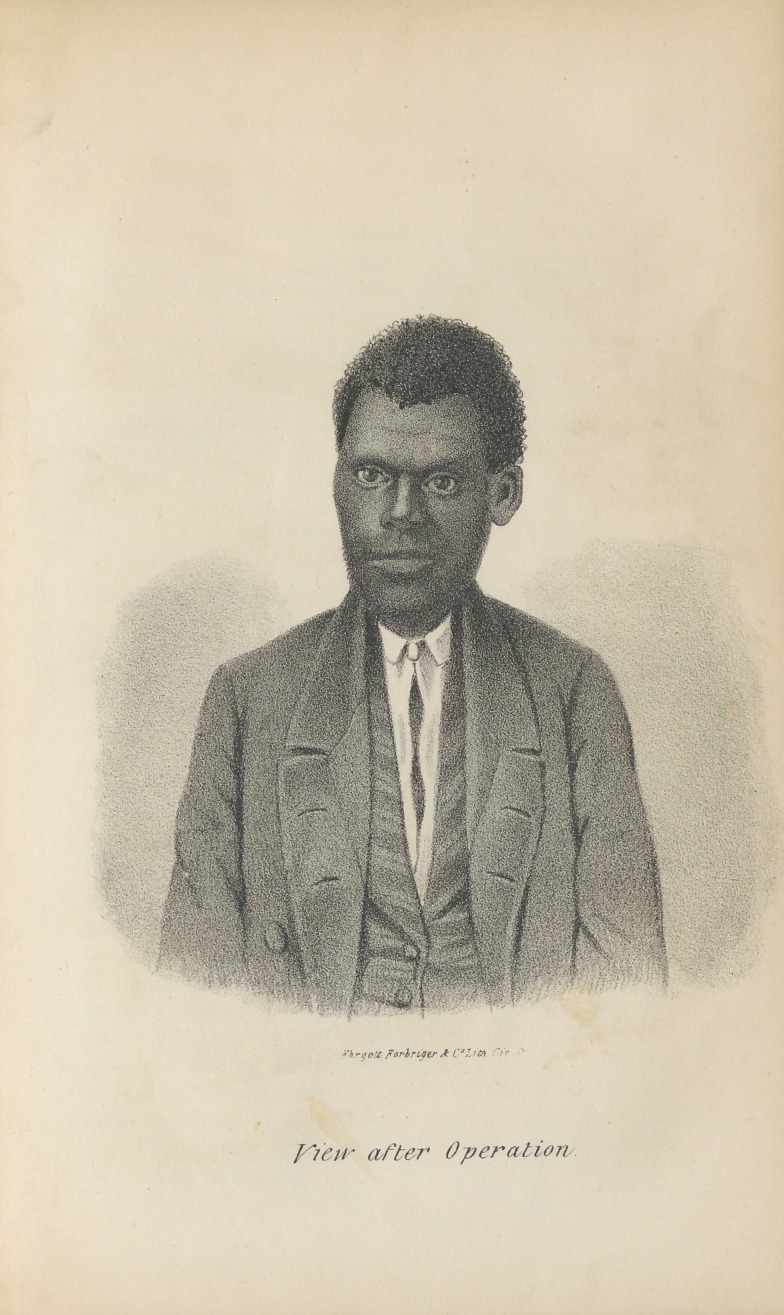# Editorial

**Published:** 1860-11

**Authors:** 


### Editorial.

### MODIFYING INFLUENCES OF DENTAL CARIES.

THE question is often asked, why is it that our teeth decay so generally and so rapidly. This is often asked, as though it could be answered by a single sentence, and it is predicated upon the supposition, that the cause is very palpable and simple, while the facts are, that the causes of caries of the teeth are numerous, diverse, and some of them not easily comprehended, at least by those who have not made the subject a matter of study and investigation. The diversity of opinion that exists in reference to this subject arises from the fact, that there has been but little general systematic investigation. While it is true that there has been some research made, in reference to the immediate exciting agents of dental caries, yet the circumstances that influence these agents, have received but little attention.

We have two or three suggestions to make, in regard to this matter, which we hope will be considered by, and elicit the attention, of all the members of our profession who feel an interest in the matter. We have reference to statistics in regard to caries of the teeth. These should have reference, we think, especially to the following things: 1st. Hereditary peculiarities; 2d. Accidental defects, and their character, either local or general; 3d. The manner of living,—the kind of food used, and the manner in which it is used; 4th. Climatic influences,—the effect of residence in malarious regions; in low marshy regions, in elevated and dry portions of the country. We think statistics in regard to the latter particular would develop some facts, that are not generally known. We shall hope to have papers from all our brethren who can give this subject their attention, and let them embody concise and complete statistics in regard to this subject. In the performance of this work let each one take a definite and well marked locality or region of country, for instance, marshy, or malarious, or dry, hilly, healthy; and if practicable, give the manner of living of the people, and all the circumstances that may be supposed to affect the teeth, either directly or indirectly. We hope these statistics will be forthcoming as soon as practicable, and we will take great pleasure in giving them to the profession. Full statistics of this kind would be an invaluable acquisition to the profession. T.

### OUR CORRESPONDENT.

In the present number, our Philadelphia Correspondent makes some strictures upon the results of the American Dental Association recently held at Washington City, in which we think he hardly does the matter justice. He seems to think that all that was accomplished was the production of four or five essays, which, upon the whole, were of very ordinary character, and not at all what the profession expected. Now, our own private opinion is, that *the* intended work of this meeting was not the production of these, or any other essays, but the work was the organization of the Association; the putting up the machinery and setting it in motion. Who ever expected a mill would grind before it was built? We do think the organization was the chief work of that meeting, and for the most part was well accomplished, and we doubt not comes up to the reasonable expectation of the profession. The principal aim of these essays was, to give variety to the exercises, rather than the production of any deep scientific researches. We think it hardly fair to hold this small and very imperfect part of the work up to view, and say that the whole was a failure. We will acquiesce in Correspondent's proposition, to await the results of another meeting,—and see if the machine can produce any better results, after it is put in motion, than before. T.

### PREPARED GUTTA PERCHA STOPPING.

We received, several months ago, a specimen of this material from the manufacturer, Dr. H. L. Jacob, of Bridgewater, England, and have since been using it in all cases where anything of the kind was indicated. It has some good qualities that we think deserve notice. It is evidently prepared with great care, the gum is very pure,—the silicious material very fine, and these are incorporated most perfectly. It is very hard, and yet very tenacious, and is of uniform consistence. In color it is made to correspond very nearly or quite to the natural teeth. It of course is a non-conductor, and is applicable in all cases where a temporary filling is desirable. It is suggested that it will answer for permanent fillings; we think, however, that cases are rare in which it should be relied upon for permanent fillings, and yet we have seen fillings, made from material much its inferior, in several respects, that have been worn for the last seven years, and that are yet apparently in good condition. It is not yet for sale in this country, but we believe arrangements are being made to supply the profession with it. T.

### NEW YORK DENTAL INSTITUTE.

The New York Journal informs us that the "New York State Dental Association" met at Saratoga, August 9th, and appointed a committee to organize a Dental Institute, draft a constitution, and report at a future meeting. We are glad to hear of any thing that looks to the improvement of the profession. We hope that, in this case, the "New York fusion" will be successful and permanent. Our brethren of the Empire State have not, hitherto, pulled well together. They have not even agreed to disagree. For example, the New York City Society appointed delegates to the National meeting, at Washington recently; but, according to the New York Journal, this move was killed by a minority, during the absence from the city of several members. Now, brethren, let us forget self, and think only of the cause in which we are engaged, and all will be well.

W.

### OBITUARY.

Death of Dr. Chapin A. Harris.—We are pained to announce the death, at his late residence in this city, of Professor Chapin A. Harris, the Father of the Science of American Dentistry, and one of the most laborious and useful professional authors, teachers and practitioners in our country. This sad event occurred on Saturday afternoon, Sept. 29th, and was the result of long illness contracted by sheer over-work in his excessive and varied labors in the line of his science, to which he was but too ardently devoted.

The career of Dr. Harris has been full of interest, and he has achieved in his thirty years of professional life, very marked results in elevating and developing the important branch of medicine, for which he has so long and so well labored. He was born in 1806, at Pompey, Onondaga county, New York, and graduated with honor as a regular physician about 1830. After practising medicine for several years, he turned his attention to dentistry, then hardly regarded as a science, and comparatively little understood as a distinct practice.

In 1840 he founded the Baltimore College of Dental Surgery, the first of its kind, we believe, in the world. Of this successful Institution he was the leading Professor und Lecturer for the twenty years, since its opening. His elaborate "Dictionary of Dental Science," 1849, and the more extended work, "Dictionary of Medicine, Dental Surgery and the Collateral Sciences," 1854, r. 8vo. pp. 800; the "Principles and Practice of Dental Surgery," 1839, eighth edition, 1838, 8vo., pp. 892, are but a few of the principal literary labors of his life. He has also translated from the French several valuable medical works, and has steadily edited since its commencement in 1839, over twenty years ago, the American Journal of Dental Science, assisted at various times by Dr. E. Parmly, S. Brown, E. Maynard, A. Westcot, W. H. Dwinelle, A. A. Blandy and A. Snowden Piggott.

We can not, in this hurried notice, undertake to do justice to the many private virtues and public services of the lamented deceased, whose death is deplored by so wide a circle of sorrowing relations and not less devoted friends. We hope hereafter to give a more worthy sketch of his career.

The above from the Baltimore American, conveys the sad intelligence of the death of Dr. Chapin A. Harris.

Dr. Harris was, probably, the oldest active member of the profession in the United States, and has certainly done more for the profession than any other man. He entered its ranks when it was young, feeble and unpopular ; he early took a high stand, and maintained his position. He as a professional man, was every way worthy of emulation. His life was no doubt much shortened, by the incessant labor which he performed for the profession.

It is useless to specify to our readers the particular object upon which he bestowed his labor, for these are well known to all the members of the profession. Notwithstanding he did so much, it was all well done, better than could have been anticipated. He was perfectly devoted to his profession, from the time he entered it till his death. The loss thus sustained is very great to the dental profession throughout the world. His memory will ever be held in grateful remembrance by those who knew him, and his name will descend through future generations as one of the chief pioneers in this specialty of medical science. T.

### TRANSACTIONS OF THE AM. DENTAL ASSOCIATION.

The proceedings of this body have been published in a volume, in which is included the Minutes of the preliminary meeting held at Niagara in August, 1859, and the Minutes of the meeting held at Washington City, D. C., on the 31st of July, 1860 ; it also includes the Constitution and By-Laws and the Essays read at the latter meeting. It has been thought best to do this, in order that the entire proceedings may be obtained together. Copies may be had upon application to either member of the Publishing Committee. T.

☞ We neglected, in the last number of the Register, to give the Cosmos credit for the cuts which illustrate that portion of the article on the fifth pair of nerves, published in that number. We were under much obligation for them, and hope for an opportunity to reciprocate. T.

## Figures and Tables

**Figure f1:**
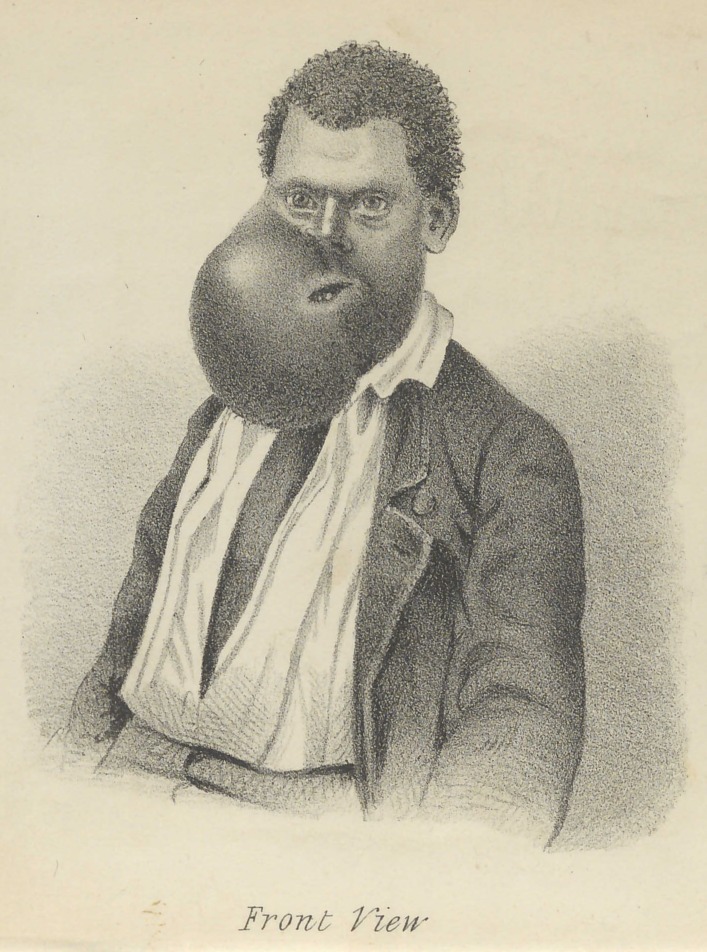


**Figure f2:**
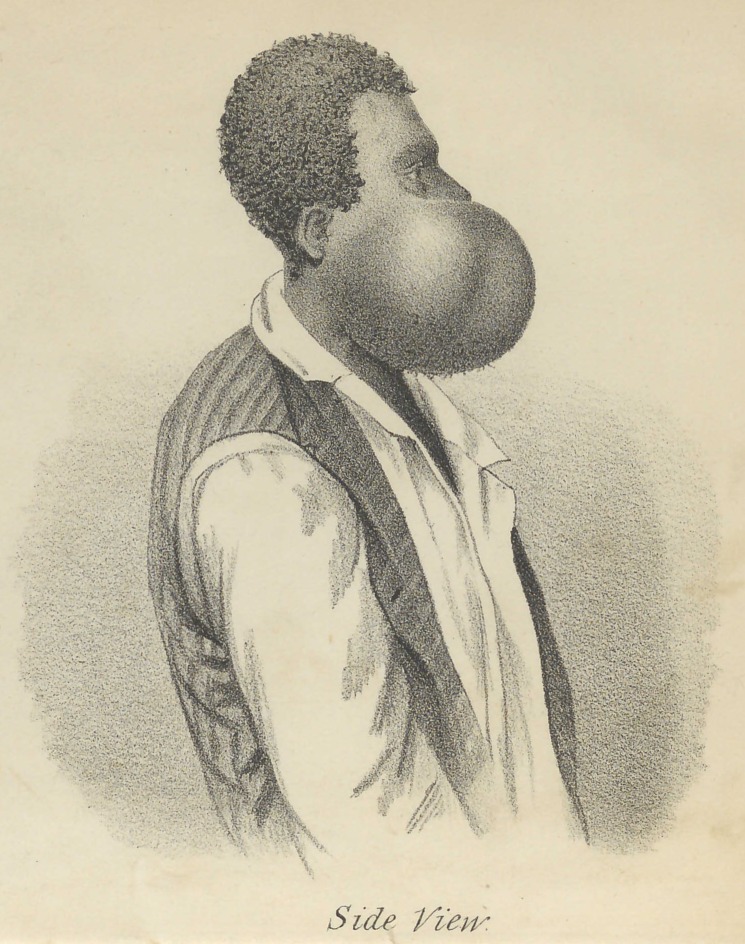


**Figure f3:**